# Synthesis, Characterization, and Electropolymerization of Extended Fused-Ring Thieno[3,4-*b*]pyrazine-Based Terthienyls

**DOI:** 10.3390/ma9060404

**Published:** 2016-05-26

**Authors:** Kristine L. Konkol, Ryan L. Schwiderski, Seth C. Rasmussen

**Affiliations:** Department of Chemistry and Biochemistry, North Dakota State University, NDSU Dept. 2735, Fargo, ND 58108-6050, USA; kristine.konkol@ndsu.edu (K.L.K.); ryan.schwiderski@ndsu.edu (R.L.S.)

**Keywords:** polythiophene, low band gap, electropolymerization

## Abstract

The synthesis, characterization, and electropolymerization of a series of extended fused-ring thieno[3,4-*b*]pyrazine-based terthienyls are reported. The target terthienyls contain a central extended thieno[3,4-*b*]pyrazine analogue containing 2-thienyl units at the reactive α-positions of the central thiophene. The extended fused-ring thieno[3,4-*b*]pyrazine analogues studied include acenaphtho[1,2-*b*]thieno[3,4-*e*]pyrazine, dibenzo[*f,h*]thieno[3,4-*b*]quinoxaline, and thieno[3′,4′:5,6]-pyrazino[2,3-*f*][1,10]phenanthroline. Comparison of the electrochemical and photophysical properties to simple thieno[3,4-*b*]pyrazine-based terthienyls and their polymeric analogues are reported in order to provide structure-function relationships within this series of compounds and materials.

## 1. Introduction

Although conjugated organic polymers are typically viewed as very modern materials, the synthesis and study of these polymers dates back to the 19th century [1,2,3,4]. Significant interest in these materials, however, did not develop until the first reports of their conductive nature in the early 1960s [5,6,7]. Advances in the 1970s then brought particular focus to these systems with the first report of metallic conductivities in conjugated polymers [8,9,10], which resulted in their inclusion in the growing field of *synthetic metals* [11]. Since then, conjugated materials have received ever increasing interest resulting from the fact that these systems combine the electronic and optical properties of classical inorganic semiconductors with many of the desirable properties of organic plastics [12,13]. This eventually resulted in the current field of organic electronics, with efforts focused on the development of technological applications such as sensors, electrochromic devices, organic photovoltaics (OPVs), organic light-emitting diodes (OLEDs), and field effect transistors (FETs) [12,13,14,15,16,17,18,19].

In attempts to optimize conjugated materials for specific applications, much effort has focused on the ability to control the polymer band gap (*E*_g_), defined as the energetic difference between the material’s filled valence and empty conduction bands [20,21,22,23]. The polymer band gap thus corresponds to the energetic separation between the highest occupied molecular orbital (HOMO) and lowest unoccupied molecular orbital (LUMO) of the bulk solid-state material and determines such properties as the lowest energy absorption and the energy of any emitted light [20,21,22,23]. In addition, as the band gap and the frontier orbitals are intimately related, control of the orbital energy levels allows tuning of both the polymer band gap and the corresponding redox properties. Such tuning of the orbital energy levels is thus also crucial in determining environmental stability, as well as proper matching of energy levels with other electronic components in device applications.

The majority of such efforts in band gap engineering of conjugated polymers has focused on producing either reduced band gap (*E*_g_ = 1.5–2.0 eV) [22,23,24,25,26,27,28] or low band gap (*E*_g_ < 1.5 eV) [21,22,23,24,25,26,27,28] polymers, largely driven by the desire to obtain materials which can more efficiently absorb solar radiation for applications in solar cell technologies (*i.e.*, OPVs). Although a number of various factors can play a role in the resulting polymer band gap [20,22], there are primarily only two general approaches for the successful production of materials with lower band gaps as defined here [20,21,22,23]. The first of these approaches was introduced in 1984 by Wudl and coworkers [29,30] with the application of fused-ring thiophenes in order to enhance the quinoidal nature of the polymer backbone. An alternate approach based on ‘donor-acceptor’ (D-A) polymeric frameworks was then introduced by Havinga and co-workers in 1992 [31,32]. Polymers based on such D-A frameworks are currently the most commonly applied approach for the production of lower *E*_g_ materials.

**Figure 1:**
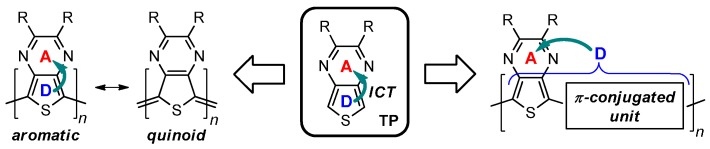
Monomeric thieno[3,4-*b*]pyrazine (TP) and TP-based materials.

A common building block utilized in the production of a wide range of both reduced and low *E*_g_ materials has been the thieno[3,4-*b*]pyrazine (TP, Figure 1) unit [23]. As outlined in Figure 1, the low *E*_g_ of TP-based materials is the result of a combination of the quinoidal character of the TP unit, as well as its strong ambipolar nature, which results in an internal intramolecular charge transfer (ICT) transition from a thiophene-localized HOMO to a more pyrazine-localized LUMO [33,34,35]. As a result, all TP-based materials exhibit properties of D-A frameworks, even simple TP homopolymers.

**Figure 2:**
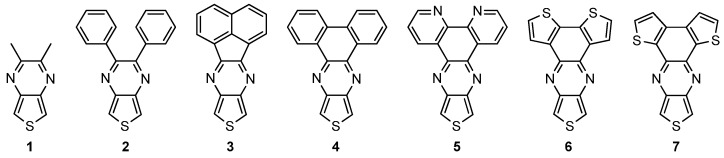
Representative thieno[3,4-*b*]pyrazines (**1**,**2**) and extended fused-ring analogues (**3**–**7**).

While the majority of TP-based materials utilize various 2,3-difunctionalized TP monomers, a number of extended fused-ring analogues (Figure 2, **3**–**7**) [36] have also been utilized as potentially improved building blocks in conjugated materials [23,37,38,39,40,41,42,43,44,45,46,47,48,49,50,51,52,53,54]. Such extended fused-ring analogues have included acenaphtho[1,2-*b*]thieno[3,4-*e*]pyrazines (**3**) [37,38,39,40,41,42,43,44,45,46,47], dibenzo[*f,h*]thieno[3,4-*b*]quinox-alines (**4**) [45,46,47,48,49,50,51], thieno[3′,4′:5,6]pyrazino[2,3-*f*][1,10]phenanthroline (**5**) [52,53], trithieno[3,4-*b*:3′,2′- *f*:2″,3″-*h*]quinoxaline (**6**) [44,54], and trithieno[3,4-*b*:2′,3′-*f*:3″,2″-*h*]quinoxaline (**7**) [44,54]. Extended fused-ring TPs are generally applied as stronger acceptors in D-A materials [47], yet these monomers are in reality ambipolar units in the same manner as traditional TPs [35]. As a result, such extended fused-ring TPs are also strong donors and exhibit characteristic internal ICT transitions [36].

**Figure 3:**
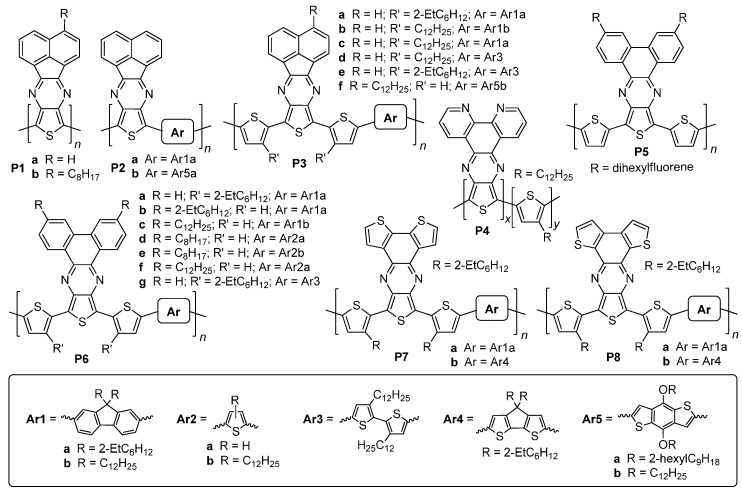
Polymeric materials incorporating extended fused-ring thieno[3,4-*b*]pyrazine analogues.

**Figure 4:**
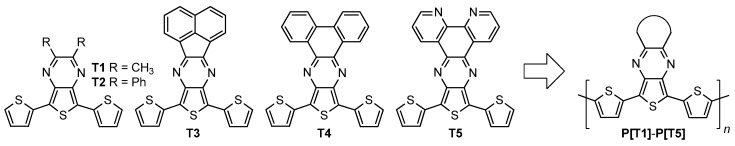
Thieno[3,4-*b*]pyrazine-based terthienyls (**T1**–**T5**) characterized in the current study and their corresponding electropolymerized polymeric materials (**P[T1]**–**P[T5]**).

Of the extended fused-ring TPs given in Figure 2, only **3** is sterically small enough to allow the production of homopolymeric materials [37,38] and thus nearly all known polymers of **3**–**7** consist of copolymeric D-A frameworks as shown in Figure 3. The extended fused-ring TP is generally incorporated into these materials as a TP-based terthienyl, which is then copolymerized with additional monomeric species (as illustrated in polymers **P3** and **P6**–**P8**). In addition to providing a structural motif that accommodates the increased size of the extended fused-ring TP, many favor TP-based terthienyls over the somewhat reactive nature of monomeric TPs [26,55]. Although the increased conjugation length of these TP-based terthienyls actually cause them to undergo oxidation at lower potentials than the analogous TP monomers, the combination of increased size and longer conjugation length results in slower reactivity and thus less production of unwanted byproducts via oxidative coupling or decomposition [26,55]. Considering the extensive use of the TP-based terthienyl motif in the materials shown in Figure 3, it is somewhat surprising that most have overly complicated polymeric structures. In fact, **P5** represents the only known example of the direct polymerization of an extended fused-ring TP-based terthienyl [51]. Of course, the majority of studies involving extended fused-ring TP-based terthienyls have focused on the resulting materials, with characterization of the TP-based terthienyls a lower priority. As such, little effort has been made to correlate the effect of chemical structure on the electronic and optical properties. In order to provide a better understanding of the structure–function relationships in such extended fused-ring TP-based terthienyls and their materials, the current report collects the complete characterization of extended TP-based terthienyls **T3**–**T5** (Figure 4), along with comparison to the simple TP analogues **T1** and **T2**. A series of simple polymeric examples of such extended fused-ring TP-based terthienyls is then reported for the first time via electropolymerization of the series **T1**–**T5**.

## 2. Results and Discussion

### 2.1. TP-Based Terthienyl Synthesis

**Scheme 1:**
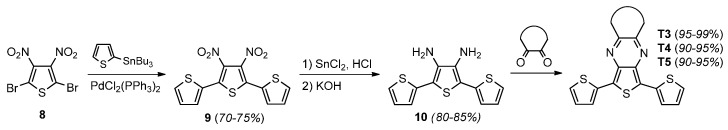
Synthesis of extended fused-ring TP-based terthienyls **T3**–**T5**.

The extended fused-ring TP-based terthienyls were synthesized by fairly standard conditions as illustrated in Scheme 1. These are the same methods previously reported for a variety of traditional TP-based terthienyls [23,55,56], as well as various extended fused-ring analogues [23,45,50,52], and involve the initial Stille cross-coupling of the common precursor 2,5-dibromo-3,4-dinitrothiophene (**8**) [57] with 2-(tributylstannyl)thiophene to generate 3′,4′-dinitro-2,2′:5′,2″-terthiophene (**9**) [55]. The critical step in this synthetic route is then reduction of **9** to the corresponding diamine **10**.

The reported reduction of **9** has been accomplished via a number of different reducing agents, including iron powder in AcOH [58,59], SnCl_2_ in HCl [45,51,56], and tin metal in HCl [55]. Although all of these conditions will successfully reduce **9**, there are significant differences in the resulting product purity. Transition metal-based reducing agents such as iron are especially problematic as the Fe(II) cations produced during the reaction coordinate quite strongly with the Lewis basic amine functionalities of aminothiophenes such as **10** [60]. As a result, such iron cations are very difficult to remove from **10**, making it extremely difficult to generate analytically pure materials. In terms of tin metal *versus* SnCl_2_, both methods work, although it has been found that the SnCl_2_ provides the most consistent results and is less affected by the form of the tin metal (powder *vs.* granules, *etc.*). The conditions reported here for the reduction of **9** are optimized in comparison to previous reports and provide higher yields of high quality product. One aspect of these optimized conditions involves the careful control of the pH during the neutralization step, which is believed to play a significant factor in the improved quality of **10**. Although the specific role of pH has not been determined, it is theorized that excess KOH generates contaminants during the neutralization which thus lowers the quality of the isolated species **10**. While the salt of **10** initially isolated from the tin reduction is often viewed as a simple HCl salt, the study of analogous salts of 3,4-diaminothiophene prepared via tin reductions have shown that these are actually complex salts containing both Cl^−^ and SnCl_6_^2^^−^ counterions [61]. The tin counterion contained in these salts is also known to react with KOH to form potassium stannate, K_2_Sn(OH)_6_, which exhibits diminished water solubility with increasing KOH concentration [62]. Thus, the application of excess KOH in the neutralization of the salt is more likely to generate amounts of low soluble K_2_Sn(OH)_6_, which can then contaminate the isolated product **10**.

It should be stressed that high purity samples of **10** are a golden yellow solid, rather than the other forms commonly reported [51,56], and give a high and narrow melting point. It should also be stressed that the stability of **10** is directly related to its purity and the material reported here is stable for several months under the right conditions. The enhanced purity of **10** as reported here is further evidenced by the ability to fully resolve the NMR signals at *ca.* 7.1 ppm, which have previously always been reported as a generic multiplet (see Supplementary Materials for details).

The final step of the production of **T3**–**T5** is then just a simple double condensation of **10** with the appropriate fused-ring α-dione in ethanol. Due to the low solubility of the products, precipitation from the hot ethanol solution occurs immediately upon formation. After collection via filtration, the products can then be purified by simple solvent washes to give high purity products in nearly quantitative yields (90%–99%). Previous reported yields of **T3**–**T5** have been in the range of 62%–83% [45,50,52], and it is our belief that the difference in the isolated yields reported here is completely a result of the higher quality of the diamine **10**.

### 2.2. UV-Vis Spectroscopy of TP-Based Terthienyls

**Figure 5:**
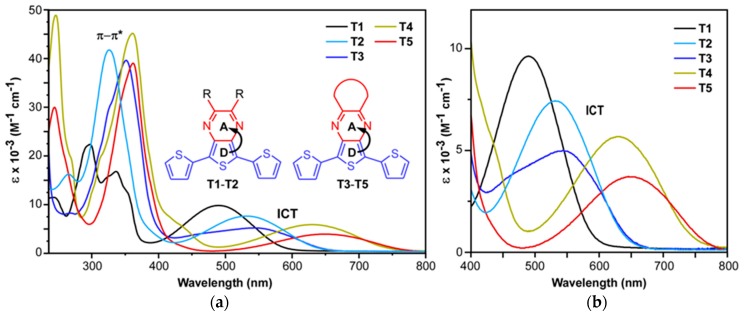
(**a**) UV-visibile spectra of TP-based terthienyls **T3**–**T5**; (**b**) visible region highlighting the ICT transitions of **T1**–**T5**.

The UV–visible spectra of the TP-based terthienyls **T1**–**T5** are shown in Figure 5 and the corresponding spectral data are given in Table 1. The absorption properties of TP-based terthienyls are characterized by a broad, low energy transition, which is formally assigned as an ICT band [55]. This assignment is consistent with the known properties of monomeric TPs, for which previous photophysical studies have revealed that the lowest energy absorption is a broad ICT band resulting from a transition between a predominately thiophene-localized HOMO and a LUMO of greater pyrazine contribution [33,34]. The addition of the 2-thienyl groups to the 5- and 7-positions of the TP unit results in a HOMO which is now delocalized across the terthienyl backbone of the TP-based terthienyls, while still retaining a LUMO dominated by pyrazine contributions [26,55]. The ICT assignment also accounts for the 138 nm red-shift observed for **T1** in comparison to the parent α-terthiophene (492 *vs.* 354 nm) [63]. As can be seen in Table 1, the extinction coefficients (ε) and oscillator strengths (*f*) of these transitions are also somewhat low. In comparison to α-terthiophene, the highest values exhibited by **T1** are still reduced nearly by half [63], which is due to the reduced “allowedness” of the ICT transition as a result of limited spatial overlap of the molecular orbitals involved [64]. The trend in the ICT transition energies for **T1**–**T5** agrees closely with the analogous monomeric extended fused-ring TPs [36], although with red shifts of 150–200 nm and nearly double the absorption intensities. The increase in extinction coefficients is consistent with the increase in cross-sectional area of the TP-based terthienyls in comparison to the monomeric analogues.

Additional stronger sets of bands are exhibited in the higher-energy region of 250–350 nm, which are assigned as localized π → π* transitions. While the extinction coefficients for these π → π* bands in simple TP-based terthienyls fall roughly between 21–35 × 10^3^ M^−1^·cm^−1^ [55], the extended fused-ring analogues exhibit higher coefficients of 39–48 × 10^3^ M^−1^·cm^−1^, with corresponding oscillator strengths of approximately 0.80–1.20. Thus, these higher energy absorptions correspond to strongly allowed transitions. The energies of these transitions agree well with the absorption energies reported for α-terthiophene [63], but also agree with localized π → π* transitions of acenapthylene [65], phenanthrene [66], and 1,10-phenanthroline [67]. As such, it is quite likely that these are not simple singular transitions for **T3**–**T5**, but the overlap of multiple localized π → π* transitions.

### 2.3. Electrochemistry of TP-Based Terthienyls

**Figure 6:**
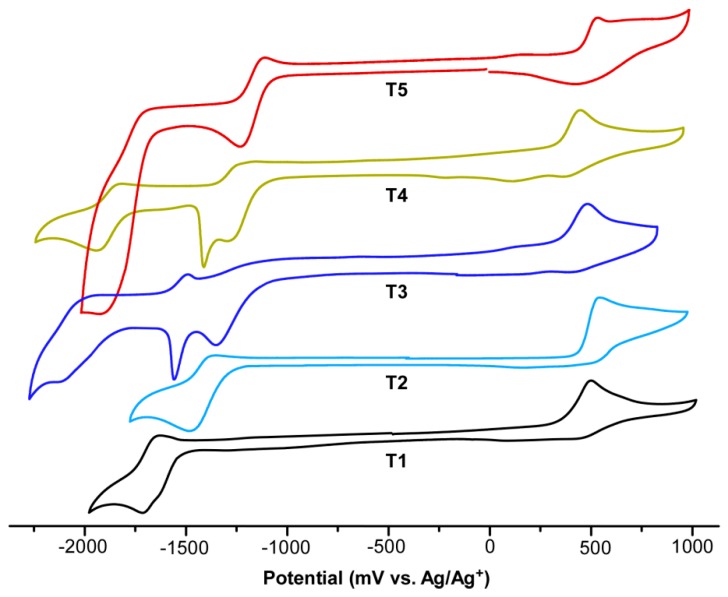
Cyclic voltammograms of thieno[3,4-*b*]pyrazine-based terthienyls **T1**–**T5**.

The electrochemical data of the TP-based terthienyls **T1**–**T5** is given in Table 2 and the cyclic voltammograms (CVs) are shown in Figure 6. Typical of previously reported TP-based terthienyls [55], **T1**–**T5** exhibit a well-defined irreversible oxidation assigned to the oxidation of the terthiophene backbone, as well as a quasireversible pyrazine-based reduction. As for most thiophene species, the irreversible nature of the oxidation here is due to the formation of thiophene-based radical cations that undergo rapid coupling to produce higher oligomeric and polymeric species. As can be seen in Table 2, the potentials of these oxidations across the series are all fairly closely spaced, a marked contrast to the more significant deviations observed for the monomer series **1**–**5** [36]. In the case of the terthienyls, the addition of the two 2-thienyl groups to the open α-positions of the central TP results in delocalization of the HOMO across the terthienyl backbone. This increased delocalization results in significant destabilization of the HOMO energy, as evidenced by a shift of 700–800 mV in the potential of oxidation from monomers **1**–**5** to the analogous terthienyls **T1**–**T5** [36]. As the central TP now only makes up one-third of the terthienyl backbone, its effect on the resulting potential of oxidation is thus significantly diminished resulting in more uniform potentials across the series. The overall trend in potentials for the two series, however, is still fairly consistent, with the most easily oxidized monomer **4** (*E*_pa_ = 0.98 V [36]) giving the most easily oxidized terthienyl **T4**.

In terms of the reduction processes of **T1**–**T5**, much greater shifts are observed across the series with an overall difference of 450 mV from the most difficult to reduce (**T1**) to the easiest (**T5**). The greater effect of TP structure on the reduction is typical of TP-based materials as the LUMO is relatively pyrazine-localized and any functionalization or ring-fusion occurs directly on the pyrazine ring [34,55]. As can been seen in Table 2 and Figure 6, the extended fused-ring analogues **T3**–**T5** undergo reduction at lower potentials than the traditional TP-based terthienyls as a result of the extended conjugation provided by the additional fused rings. This additional conjugation and the lower LUMO levels also results in the observation of two distinct reduction processes within the solvent window for **T3**–**T5**. Within these three examples, the central extended TP of **T3** has two fewer π-electrons relative to **T4** or **T5** and thus exhibits a slightly destabilized LUMO in comparison. Although **T5** is isostructural to **T4**, the additional nitrogens within the fused phenanthroline further stabilizes the LUMO of **T5** to give the lowest reduction potential of the series. 

It should be pointed out that terthienyls **T3** and **T4** exhibit adsorption waves directly following the inital reduction process, which appear as sharper “spikes” within the CV [69]. Such adsorption waves are the result of analyte strongly adsorbing to the electrode, which is most likely facilitated here by the large planar structures of the extended fused-ring terthienyls. As the adsorbed analyte is stabilized by the interaction with the electrode, it is more difficult to reduce than the material in solution, and thus the adsorption wave occurs at greater negative potential than the diffusion controled process [69]. As these adsorption waves are not observed in **T5**, it appears that the additional nitrogen content of this terthienyl inhibits this adsorption to the electrode.

### 2.4. Electropolymerization of TP-Based Terthienyls and Polymer Electrochemistry

While most current efforts focus on the production of soluble, processible materials via various organometallic-catalyzed cross-coupling methods, the electropolymerization of heterocycles as de-veloped by Art Diaz [3] provided a popular method for the production of conjugated polymer films during the rapid growth of the field in the 1980s. Although the applications of electropolymerized films are somewhat limited, the ability to produce polymer films onto electroactive species by this method provides a simple means for the electrochemical and optical characterization of the resulting films [26,70,71,72]. More importantly, this also allows the direct comparison of a series of related materials while minimizing the effects of differences in solubility or processibility. For these reasons, the electropolymerization of **T1**–**T5** was investigated in order to provide baseline reference data for the simplest examples of polymeric materials containing extended fused-ring TP-based terthienyls.

**Figure 7:**
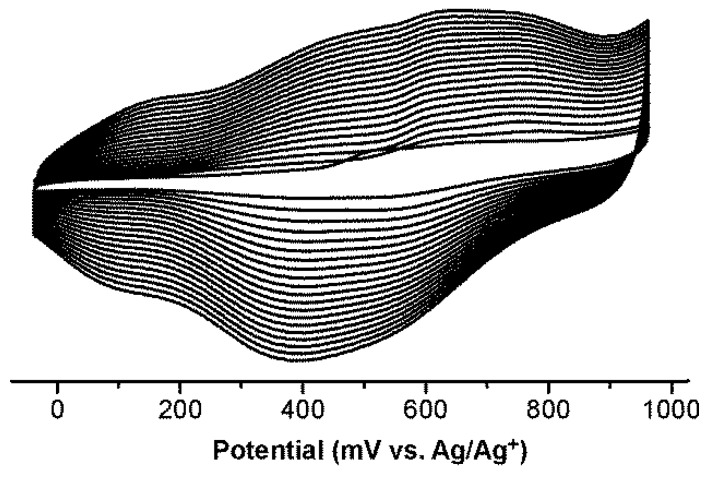
Growth of polymer **P[T5]** with potential cycling.

Potential cycling through the irreversible oxidation of **T1**–**T5** resulted in incremental growth of the corresponding conjugated polymer film on the surface of the working electrode. As shown in Figure 7 for the electropolymerization of **T5**, the oxidation wave of the initial terthienyl shifts to lower potential with an increase in current corresponding to the growth of the polymer film. The resulting polymer film thus exhibits multiple oxidative waves with the lowest potential *E*_pa_ shifted by *ca.* 360 mV in comparison to the initial terthienyl. It should be pointed out that this is in marked contrast to that seen for the electropolymerization of simple TP-based terthienyls such as **T1**, which exhibit shifts in the oxidation onset to lower potentials, but no real significant shift in the polymer *E*_pa_ [26,56]. This suggests that the polymer films of **P[T3]**–**P[T5]** contain fewer segments of lower conjugation length in comparison to the films of **P[T1]**.

**Figure 8:**
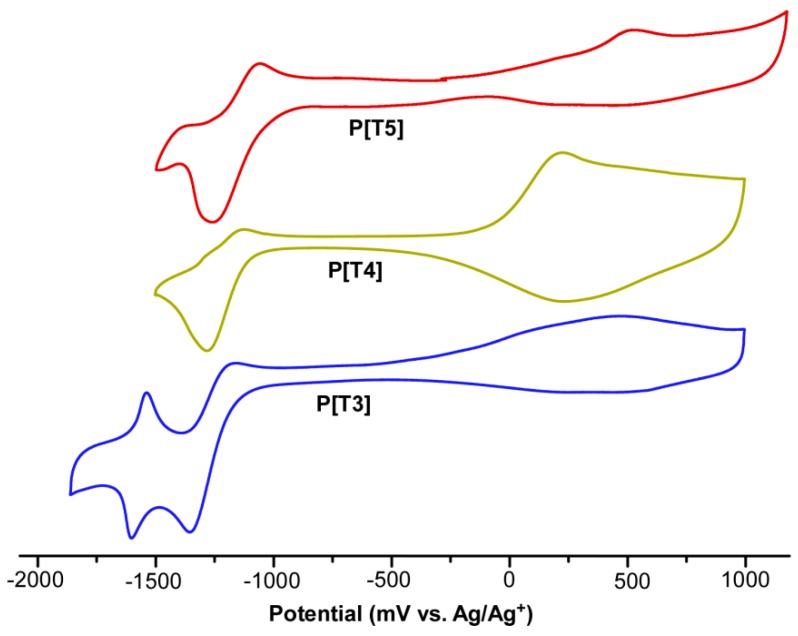
Cyclic voltammograms of extended fused-ring TP polymers **P[T3]**–**P[T5]**.

The electrochemical data for the resulting electropolymerized polymers **P[T1]**–**P[T5]** are collected in Table 3 and representative CVs are shown in Figure 8. As typical of most TP-based materials, the polymer films of the electropolymerized terthienyls exhibit amphoteric redox properties consisting of relatively broad oxidation waves with sharper, more well-defined reductions. As can be seen in Table 3, the peak potentials for both the oxidation (*E*_pa_) and reduction (*E*_pc_) processes follow the same basic trends seen in both the monomers [36] and terthienyls as given above in Table 2. However, it should be noted that the onset of oxidation for polymer **P[T4]** is quite sharp, resulting in a determined HOMO energy level that is deeper than expected. The sharper onset and more defined oxidation peak may be indicative of reduced polydispersity, with fewer segments of higher conjugation lengths in the resulting polymer film.

With the exception of the HOMO level of **P[T4]**, all of the HOMO and LUMO energy levels given in Table 3 follow expected trends, which results in band gaps of 0.8–0.9 eV for the polymers of the extended fused-ring TP analogues. It should be noted that the E_g_ values of **P[T3]**–**P[T5]** given here are lower than that reported for all of the copolymeric materials given in Figure 3 [23,40,41,42,43,44,45,46,47,48,51,53,54], with only the two homopolymers of acenaphtho[1,2-*b*]thieno[3,4-*e*]pyrazine (**P1a**, **P1b**) giving lower band gaps [23,37,38]. This is consistent with previous reports of TP-based materials which have shown that TP and its extended fused-ring analogues are stronger donors than nearly all other commonly applied building blocks [25,28,35] and thus the inclusion of additional monomeric units stabilizes the polymer HOMO and leads to an increase in *E*_g_. The examples reported here limit the inclusion of other monomers to just the two flanking thiophenes, thus increasing the overall TP content in the backbone to give higher lying HOMO levels and lower *E*_g_ values.

### 2.5. Optical Absorption of Polymer Films

**Figure 9:**
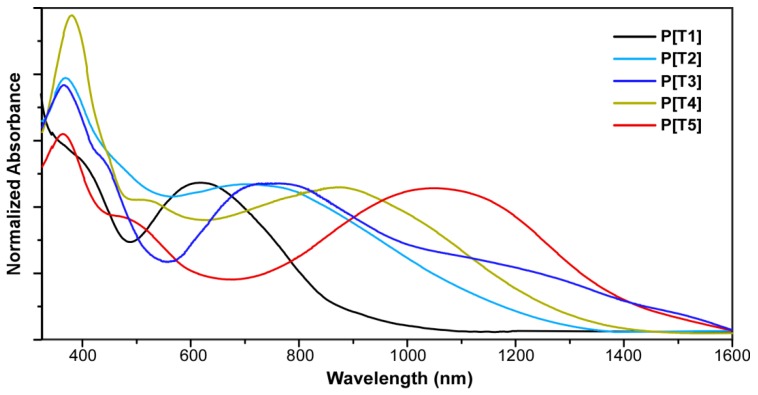
Thin film visible-NIR absorption spectra for polymers **P[T1]**–**P[T5]**.

The extended fused-ring terthienyls **T1**–**T5** were then electropolymerized onto ITO (indium tin oxide) slides, thus allowing measurement of the absorption spectra of the corresponding polymers as thin films. The collected optical data is given in Table 4 with the corresponding thin film spectra shown in Figure 9. In all cases, the polymers exhibit a low energy ICT transition in the visible to the NIR (near infrared) region of the spectrum, along with a second transition in the high energy region of the visible spectrum. The band gap values determined from the absorption onsets show good agreement with the trends found in the optical characterization of the terthienyl precursors above (Table 1 and Figure 5). In the case of **P[T3]**, electropolymerization onto ITO resulted in a quite granular deposit, rather than a smooth polymer film as desired. As such, the absorption spectrum exhibits significant scatter evidenced by the long tailing in Figure 9. Ignoring the tailing and determining the onset by extrapolation of the low energy slope of the peak at 750 nm provided an *E*_g_ value of 0.99 eV, which seems to fit the trends previously observed in the terthienyl percursors. The polymers **P[T4]** and **P[T5]** exhibit greater differences in absorption and *E*_g_ than might be expected from the optical data of **T4** and **T5**. However, if **P[T4]** contains segments of lower conjugation lengths as proposed above, this could account for the differences observed here for the absorption properties of the polymer films.

As noted with the electrochemical band gaps, the optical band gaps of 0.82–0.99 eV for **P[T3]**–**P[T5]** are lower than that reported for all of the copolymeric materials given in Figure 3 [23,40,41,42,43,44,45,46,47,48,51,53,54], which reinforces the assertion that the inclusion of additional monomer units can allow more soluble or processible films and can generate deeper HOMO levels, but this always comes at the cost of increased band gaps. Lastly, comparison of **P[T4]** with its previously reported analogue **P5** (Figure 3) reveals a significant difference in *E*_g_ (0.89 *vs.* 1.21 eV [51]). The higher *E*_g_ value reported for **P5** suggests that either the two dihexylfluorene groups appended to the central TP analogue **4** provide significant steric hindrance which reduces planarity and limits conjugation, or that **P5** is of limited molecular weight.

## 3. Materials and Methods 

The reference TP-based terthienyls 2,3-dimethyl-5,7-bis(2-thienyl)thieno[3,4-*b*]pyrazine (**T1**) and 2,3-diphenyl-5,7-bis(2-thienyl)thieno[3,4-*b*]pyrazine (**T2**) were prepared as previously reported [55]. 3′,4′-Dinitro-2,2′:5′,2″-terthiophene (**9**) was prepared from 2,5-dibromo-3,4-dinitrothiophene (**8**) [56] as previously reported [55]. CH_2_Cl_2_ and CH_3_CN were dried over CaH_2_ and distilled prior to use. All other materials were reagent grade and used without further purification. All reactions were carried out under a nitrogen atmosphere. All glassware was oven-dried, assembled hot, and cooled under a dry nitrogen stream before use. ^1^H and ^13^C NMR spectra were obtained in CDCl_3_ on a 400 MHz spectrometer (Bruker Corporation, Pittsburgh, PA, USA) and referenced to the CHCl_3_ signal. Peak multiplicity is reported as follows: s = singlet, d = doublet, dd = doublet of doublets, dt = doublet of triplets, m = multiplet and br = broad. High resolution mass spectrometry (HRMS) was obtained on a mass spectrometer (Waters, Boston, MA, USA) with electrospray ionization and quantitative time of flight.

### 3.1. Synthesis of 3′,4′-Diamino-2,2′:5′,2″-terthiophene (**10**)

The following is a modification of previously reported methods [55]. Precursor **9** (11.4 g, 33.7 mmol) and SnCl_2_ (57.5 g, 303 mmol) were added to a flask equipped with a condenser, followed by ethanol (150 mL) and HCl (150 mL). The flask was evacuated and backfilled with N_2_, after which the suspension was heated at reflux with stirring overnight. The solution was cooled to 0 °C, filtered, and washed with diethyl ether to give a tan-green solid. This salt was then added to 100 mL H_2_O at 0 °C and 1 M KOH solution added dropwise until the solution turned basic (pH 7–8). The product was extracted from the basic solution with CH_2_Cl_2_, and the combined organic extracts were washed with H_2_O, dried over MgSO_4_, and the solvent was removed via rotatory evaporation to yield a golden yellow solid (80%–85%) (***Note***: If stored in the dark at reduced temperature, **10** is stable for a period of several months). mp 97.5–98.6 °C (lit. mp 96.0–96.5 °C [56]); ^1^H NMR (CDCl_3_) δ 7.27 (dd, *J* = 1.4, 4.8 Hz, 2H), 7.10 (dd, *J* = 1.4, 3.7 Hz, 2H), 7.08 (dd, *J* = 3.7, 4.8 Hz, 2H), 3.67 (br s, 4H); ^13^C NMR (CDCl_3_) δ 136.0, 133.6, 127.8, 124.0, 123.9, 110.2. NMR spectra are provided in the Supplementary Materials and all NMR data agree well with previously reported values [59].

### 3.2. General Synthesis of Extended Fused-Ring Thieno[3,4-b]pyrazine-based Terthienyls

Diamine **10** (278 mg, 1.00 mmol) and the appropriate fused-ring dione (1.20 mmol) were added to a flask equipped with a condenser, which was then evacuated and backfilled with N_2_ three times. Absolute EtOH (50 mL) was then added and the mixture heated to reflux with stirring until a colored precipitate formed (6 h to overnight). The reaction was then cooled to room temperature and filtered. The collected precipitate was washed well with first ethanol and then hexanes to yield **T3**–**T5** as analytically pure colored solids.

#### 3.2.1. 8,10-Bis(2-thienyl)acenaphtho[1,2-*b*]thieno[3,4-*e*]pyrazine (**T3**)

Terthienyl **T3** was obtained as a dark purple solid (95%–99% yield). mp ~330 °C (dec); ^1^H NMR (CDCl_3_, 50 °C) δ 8.37 (d, *J* = 7.1 Hz, 2H), 8.08 (d, *J* = 8.3 Hz, 2H), 7.83 (dd, *J* = 7.1, 8.3 Hz, 2H), 7.78 (dd, *J* = 1.2, 3.6 Hz, 2H), 7.44 (dd, *J* = 1.2, 5.1 Hz, 2H), 7.17 (dd, *J* = 3.6, 5.1 Hz, 2H). The ^1^H NMR spectrum is provided in the Supplementary Materials and all NMR data agree well with previously reported values [45]; ^13^C NMR not obtained due to low solubility; HRMS *m*/*z* 424.0189 [M+] (calcd for C_24_H_12_N_2_S_3_ 424.0163).

#### 3.2.2. 10,12-Bis(2-thienyl)dibenzo[*f,h*]thieno[3,4-*b*]quinoxaline (**T4**)

Terthienyl **T4** was obtained as a dark green solid (90%–95% yield). mp ~290 °C (dec); ^1^H NMR (CDCl_3_, 50 °C) δ 9.32 (dd, *J* = 2.0, 7.8 Hz, 2H), 8.45 (dd, *J* = 1.4, 7.8 Hz, 2H), 7.77 (dd, *J* = 1.0, 3.7 Hz, 2H), 7.75 (dt, *J* = 2.0, 7.8 Hz, 2H), 7.71 (dt, *J* = 1.4, 7.8 Hz, 2H), 7.49 (dd, *J* = 1.0, 5.2 Hz, 2H), 7.19 (dd, *J* = 3.7, 5.2 Hz, 2H). The ^1^H NMR spectrum is provided in the Supplementary Materials; ^13^C NMR not obtained due to low solubility; HRMS *m*/*z* 450.0320 [M+] (calcd for C_26_H_14_N_2_S_3_ 450.0319).

#### 3.2.3. 10,12-Bis(2-thienyl)thieno[3′,4′:5,6]pyrazino[2,3-*f*][1,10]phenanthroline (**T5**)

Terthienyl **T5** was obtained as a dark turquoise solid (90%–95% yield). mp ~360 °C (dec); ^1^H NMR (CDCl_3_, 50 °C) δ 9.15 (dd, *J* = 2.0, 8.0 Hz, 2H), 9.10 (dd, *J* = 2.0, 4.3 Hz, 2H), 7.55 (dd, *J* = 4.3, 8.0 Hz, 2H), 7.47 (dd, *J* = 1.1, 3.7 Hz, 2H), 7.40 (dd, *J* = 1.1, 5.1 Hz, 2H), 7.08 (dd, *J* = 3.7, 5.1 Hz, 2H). The ^1^H NMR spectrum is provided in the Supplementary Materials; ^13^C NMR not obtained due to low solubility; HRMS *m*/*z* 453.0316 [M + H]^+^ (calcd for C_24_H_12_N_4_S_3_H 453.0302).

### 3.3. Electropolymerizations

Electropolymerizations of **T1**–**T5** were carried out in a three-electrode cell consisting of a platinum disc working electrode, a platinum wire auxiliary electrode, and Ag/Ag^+^ reference electrode. Solutions consisted of oligomer dissolved in anhydrous CH_3_CN or CH_2_Cl_2_ containing 0.10 M tetrabutylammonium hexafluorophosphate (TBAPF_6_). The solutions were deoxygenated by sparging with argon prior to each scan and blanketed with argon during the polymerizations. The platinum disc working electrode was polished with 0.05 mm alumina and washed well with deionized water and dry solvent prior to each film growth. The films were grown by cyclic voltammetry scanning through the *E*_pa_ region for each oligomer.

Electropolymerizations for optical experiments were carried out in the same manner as discussed above except an ITO-coated glass plate was used as the working electrode. Polymer films were grown by continuous repeated potential cycling around the *E*_pa_ for each monomer until a suitable film was obtained and then held at a fixed potential corresponding to the neutral form of the polymer under investigation.

### 3.4. Electrochemical Measurements

All electrochemical methods were performed utilizing a three-electrode cell consisting of platinum disc working electrode, a platinum wire auxiliary electrode, and a Ag/Ag^+^ reference electrode (0.251 V *vs.* saturated calomel electrode (SCE)) [73]. Supporting electrolyte consisted of 0.10 M TBAPF_6_ in dry CH_3_CN or CH_2_Cl_2_. Solutions were deoxygenated by sparging with argon prior to each scan and blanketed with argon during the measurements. All measurements were collected at a scan rate of 100 mV/s.

### 3.5. Absorption Spectroscopy

UV-visible spectra of **T1**–**T5** were measured on a dual beam scanning spectrophotometer (Varian, Mulgrave, Australia) using samples prepared as dilute CH_2_Cl_2_ or CH_3_CN solutions in 1 cm quartz cuvettes. Oscillator strengths were determined from the visible spectra via spectral fitting to accurately quantify the area of each transition and then calculated using literature methods [74]. Visible-NIR spectra of the polymeric materials were measured as polymer films on ITO-coated glass plates. 

## 4. Conclusions

The optical and electrochemical characterization of TP-based terthienyls **T3**–**T5** containing the extended fused-ring units acenaphtho[1,2-*b*]thieno[3,4-*e*]pyrazine (**3**), dibenzo[*f,h*]thieno[3,4-*b*]quin-oxaline (**4**), and thieno[3′,4′:5,6]pyrazino[2,3-*f*][1,10]phenanthroline (**5**) have revealed that these terthienyl species follow the same trends in properties as the respective monomeric units **3**–**5**. In a similar manner, the electropolymerization of these terthienyls provide low band gap materials (*E*_g_ = 0.8–1.0 eV) whose polymer films exhibit optical and electronic properties consistent with the trends of both the TP-based terthienyls **T3**–**T5** and the initial monomeric TP analogues **3**–**5**. The fact that these trends hold from monomer to terthienyl to polymer reinforces the claim that the ambipolar nature of TPs and their extended analogues contributes fully to both the HOMO and LUMO of any resulting TP-based materials. As a result, it is thus the electronic nature of the ambipolar TP unit that provides the greatest contribution to the determination of the frontier orbital energies and band gap of TP-based materials.

## Figures and Tables

**Table 1:** UV-visible spectral data for thieno[3,4-*b*]pyrazine-based terthienyls **T1**–**T5**. ^1^

Terthienyl	S_0_ → S_1_ (ICT)			S_0_ → S_2_ (π → π*)		
	λ_ma_ (nm)	ε (M^−1^·cm^−1^)	*f*	λ_max_ (nm)	ε (M^−1^·cm^−1^)	*f*
T1 ^2^	492	12100	0.242	339	21000	0.268
T2 ^2^	540	7800	0.123	338	45100	0.908
T3	547	5000	0.139	352	40400	0.822
T4	629	5800	0.097	361	47500	1.183
T5	644	3800	0.060	360	39300	0.793

^1^ In CH_2_Cl_2_; S_0_ = singlet ground state; S_1_ = first singlet excited state; S_2_ = second singlet excited state; ICT = intramolecular charge transfer; λ_max_ = wavelength of absorbance maximum; ε = extinction coefficient; *f* = oscillator strength; ^2^ Ref. [55], In CH_3_CN.

**Table 2:** Electrochemical data for thieno[3,4-*b*]pyrazine-based terthienyls **T1**–**T5**. ^1^

Terthienyl	Oxidation	Reduction				*E*_HOMO_ (eV) ^2^	*E*_LUMO_ (eV) ^3^
	*E*_pa_ (V)	*E*_½_ (red1, V)	Δ*E* (mV)	*E*_pc_ (red2, V)	Δ*E* (mV)		
**T1** ^4^	0.50	−1.68	100	-	-	−5.39	−3.46
**T2** ^4^	0.54	−1.46	80	-	-	−5.41	−3.80
**T3**	0.49	−1.35 ^5^	-	−2.55	100	−5.37	−3.82
**T4**	0.43	−1.28	80	−1.90	120	−5.29	−3.86
**T5**	0.51	−1.23	110	−1.82	130	−5.40	−3.91

^1^ All potentials *vs.* Ag/Ag^+^. In CH_2_Cl_2_ containing 0.10 M TBAPF_6_; *E*_pa_ = aniodic peak potential; *E*_½_ = half-wave potential; Δ*E* = peak separation; *E*_pc_ = cathodic peak potential; ^2^ *E*_HOMO_ = −[*E*_(onset,ox *vs.* ferrocenium/ferrocene)_ + 5.1] (eV), Ref. [68]; ^3^: *E*_LUMO_ = −[*E*_(onset,red *vs.* ferrocenium/ferrocene)_ + 5.1) (eV), Ref. [68]; ^4^ Ref. [55], 0.10 M TBAPF_6_ in CH_3_CN; ^5^ Irreversible, *E*_pc_ reported.

**Table 3:** Electrochemical data for polymer films of **P[T1]**–**P[T5]**. ^1^

Polymer	Oxidation		Reduction		*E*_HOMO_ (eV) ^2^	*E*_LUMO_ (eV) ^3^	*E*_g_^elec^ (eV)
	*E*_pa_ (V)	*E*_onset_ (V)	*E*_pc_ (V)	*E*_onset_ (V)			
**P[T1]** ^4^	0.54, 0.85	0.32	−1.48, −1.82	−1.24	−5.37	−3.81	1.56
**P[T2]** ^4^	0.58	0.33	−1.39	−1.00	−5.38	−4.05	1.33
**P[T3]**	0.45	−0.25	−1.37, −1.63	−1.05	−4.80	−4.00	0.80
**P[T4]**	0.24	−0.07	−1.28	−0.95	−4.98	−4.10	0.88
**P[T5]**	0.51	0.00	−1.25	−0.90	−5.05	−4.15	0.90

^1^ All potentials *vs.* Ag/Ag^+^. In CH_2_Cl_2_ containing 0.10 M TBAPF_6_; *E*_pa_ = aniodic peak potential; *E*_onset_ = onset potential; *E*_pc_ = cathodic peak potential; *E*_g_^elec^ = electrochemical band gap; ^2^ *E*_HOMO_ = −[*E*_(onset,ox *vs.* ferrocenium/ferrocene)_ + 5.1] (eV), Ref. [68]; ^3^ *E*_LUMO_ = − [*E*_(onset,red *vs.* ferrocenium/ferrocene)_ + 5.1] (eV), Ref. [68]; ^4^ Ref. [26], 0.10 M TBAPF_6_ in CH_3_CN.

**Table 4:** Absorption data for polymer films of **P[T1]**–**P[T5]**. ^1^

Polymer	λ_max_ (nm)	λ_onset_ (nm)	*E*_g_^opt^ (eV)
**P[T1]** ^2^	626	850	1.46
**P[T2]** ^1^	712	1230	1.01
**P[T3]**	750	1250	0.99
**P[T4]**	880	1400	0.89
**P[T5]**	1060	1510	0.82

^1^ Measured as films electropolymerized onto ITO slides; λ_max_ = wavelength of absorbance maximum; λ_onset_ = wavelength of absorbance onset; *E*_g_^opt^ = optical band gap; ^2^ Ref. [26].

## Supplementary Materials

The following are available online at www.mdpi.com/1996-1944/9/6/404/s1. Figure S1: ^1^H NMR Spectrum of Compound **10**; Figure S2: ^13^C NMR Spectrum of Compound **10**, Modeling of 2nd order coupling effects in Compound **10**; Figure S3: Comparison of results from modeling of 2nd order coupling effects in the ^1^H NMR spectrum of **10**; Figure S4: ^1^H NMR Spectrum of Compound **T3**; Figure S5: ^1^H NMR Spectrum of Compound **T4**; Figure S6: ^1^H NMR Spectrum of Compound **T5**.

## Abbreviations

The following abbreviations are used in this manuscript:

Δ*E*	=peak separation
ε	extinction coefficient
*f*	=oscillator strength
λ_max_	wavelength of absorbance maximum
D-A	donor-acceptor
*E*_g_	band gap
*E*_g_^elec^	electrochemical band gap
*E*_g_^opt^	optical band gap
*E*_onset_	onset potential
*E*_pa_	aniodic peak potential
*E*_pc_	cathodic peak potential
*E*_½_	half-wave potential
HOMO	highest occupied molecular orbital
HRMS	high resolution mass spectrometry
ICT	intramolecular charge transfer
ITO	indium tin oxide
lit.	literature
LUMO	lowest unoccupied molecular orbital
mp	melting point
NIR	near infrared
NMR	nuclear magnetic resonance
OLEDs	organic light-emitting diodes
OPVs	organic photovoltaics
S_0_	singlet ground state
S_1_	first singlet excited state
S_2_	second singlet excited state
SCE	saturated calomel electrode
TBAPF_6_	tetrabutylammonium hexafluorophosphate
TP	thieno[3,4-*b*]pyrazineReferences

## References

[1] Rasmussen S.C., Strom E.T., Rasmussen S.C. (2011). Electrically conducting plastics: Revising the history of conjugated organic polymers. 100+ Years of Plastics. Leo Baekeland and Beyond.

[2] Rasmussen S.C. (2014). The path to conductive polyactylene. Bull. Hist. Chem..

[3] Rasmussen S.C. (2015). Early history of polypyrrole: The first conducting organic polymer. Bull. Hist. Chem..

[4] Rasmussen S.C., Zhang Z., Rouabhia M., Moulton S. (2016). Early history of conductive organic polymers. Conductive Polymers: Electrical Interactions in Cell Biology and Medicine.

[5] McNeill R., Siudak R., Wardlaw J.H., Weiss D.E. (1963). Electronic conduction in polymers. I. The chemical structure of polypyrrole. Aust. J. Chem..

[6] Bolto B.A., Weiss D.E. (1963). Electronic conduction in polymers. II. The electrochemical reduction of polypyrrole at controlled potential. Aust. J. Chem..

[7] Bolto B.A., McNeill R., Weiss D.E. (1963). Electronic conduction in polymers. III. Electronic properties of polypyrrole. Aust. J. Chem..

[8] Shirakawa H., Louis E.J., MacDiarmid A.G., Chiang C.K., Heeger A.J. (1977). Synthesis of Electrically conducting organic polymers: Halogen derivatives of polyacetylene, (CH)_x_. Chem. Soc. Chem. Commun..

[9] Chiang C.K., Fincher C.R., Park Y.W., Heeger A.J., Shirakawa H., Louis B.J., Gau S.C., MacDiarmid A.G. (1977). Electrical conductivity in doped polyacetylene. Phys. Rev. Lett..

[10] Chiang C.K., Druy M.A., Gau S.C., Heeger A.J., Louis E.J., MacDiarmid A.G., Park Y.W., Shirakawa H. (1978). Synthesis of Highly Conducting Films of Derivatives of Polyacetylene, (CH)_x_. J. Am. Chem. Soc..

[11] Rasmussen S.C. (2016). On the origin of ‘synthetic metals’. Mater. Today.

[12] Skotheim T.A., Reynolds J.R., Skotheim T.A., Reynolds J.R. (2007). Handbook of Conducting Polymers.

[13] Perepichka I.F., Perepichka D.F., Perepichka I.F., Perepichka D.F. (2009). Handbook of Thiophene-Based Materials.

[14] Perepichka I.F., Perepichka D.F., Meng H., Wudl F. (2005). Light-emitting polythiophenes. Adv. Mater..

[15] Günes S., Neugebauer H., Sariciftci N.S. (2007). Conjugated polymer-based organic solar cells. Chem. Rev..

[16] Grimsdale A.C., Chan K.L., Martin R.E., Jokisz P.G., Holmes A.B. (2009). Synthesis of light-emitting conjugated polymers for applications in electroluminescent devices. Chem. Rev..

[17] Scharber M.C., Sariciftci N.S. (2013). Efficiency of bulk-heterojunction organic solar cells. Prog. Polym. Sci..

[18] Nielsen C.B., McCulloch I. (2013). Recent advances in transistor performance of polythiophenes. Prog. Polym. Sci..

[19] Rasmussen S.C., Evenson S.J., McCausland C.B. (2015). Fluorescent thiophene-based materials and their outlook for emissive applications. Chem. Commun..

[20] Rasmussen S.C., Ogawa K., Rothstein S.D., Nalwa H.S. (2008). Synthetic approaches to band gap control in conjugated polymeric materials. Handbook of Organic Electronics and Photonics.

[21] Rasmussen S.C., Pomerantz M., Skotheim T.A., Reynolds J.R. (2007). Low bandgap conducting polymers. Handbook of Conducting Polymers.

[22] Rasmussen S.C., Muellen K., Kobayashi S. (2015). Low-bandgap polymers. The Encyclopedia of Polymeric Nanomaterials.

[23] Rasmussen S.C., Schwiderski R.L., Mulholland M.E. (2011). Thieno[3,4-*b*]pyrazines and their applications to low band gap organic materials. Chem. Commun..

[24] Mulholland M.E., Schwiderski R.L., Rasmussen S.C. (2012). Structure-function relationships in conjugated materials containing tunable thieno[3,4-*b*]pyrazine units. Polym. Bull..

[25] Mulholland M.E., Schwiderski R.L., Evenson S.J., Rasmussen S.C. (2012). Molecular design of conjugated materials: Applications of tunable, ambipolar Thieno[3,4-*b*]pyrazine building blocks. Polym. Mater. Sci. Eng..

[26] Schwiderski R.L., Rasmussen S.C. (2014). Side chain tuning of frontier orbitals in polymers of thieno-[3,4-*b*]pyrazine-based terthienyls. Synth. Met..

[27] Mulholland M.E., Wen L., Rasmussen S.C. (2015). Dialkyl- and dialkoxy-functionalized Poly(thieno[3,4-*b*]-pyrazine)s via GRIM polymerization: Side chain tuning of electronic and optical properties. Topol. Supramol. Polym. Sci..

[28] Mulholland M.E., Konkol K.L., Anderson T.E., Schwiderski R.L., Rasmussen S.C. (2015). Tuning the light absorption of donor-acceptor conjugated polymers: Effects of side chains and ‘spacer’ units in thieno[3,4-*b*]pyrazine-flourene copolymers. Aust. J. Chem..

[29] Wudl F., Kobayashi M., Heeger A.J. (1984). Poly(isothianaphthene). J. Org. Chem..

[30] Kobayashi M., Colaneri N., Boysel M., Wudl F., Heeger A.J. (1985). The electronic and electrochemical properties of poly(isothianaphthene). J. Chem. Phys..

[31] Havinga E.E., ten Hoeve W., Wynberg H. (1992). A new class of small band gap organic polymer conductors. Polym. Bull..

[32] Havinga E.E., ten Hoeve W., Wynberg H. (1993). Alternate donor-acceptor small-band-gap semiconducting polymers; Polysquaraines and polycroconaines. Synth. Met..

[33] Rasmussen S.C., Sattler D.J., Mitchell K.A., Maxwell J. (2004). Photophysical characterization of 2,3-difunctionalized thieno[3,4-*b*]pyrazines. J. Lumin..

[34] Wen L., Nietfeld J.P., Amb C.M., Rasmussen S.C. (2008). Synthesis and characterization of new 2,3-disubstituted thieno[3,4-*b*]pyrazines: Tunable building blocks for low band gap conjugated materials. J. Org. Chem..

[35] Wen L., Heth C.L., Rasmussen S.C. (2014). Thieno[3,4-*b*]pyrazine-based oligothiophenes: Simple models of donor-acceptor polymeric materials. Phys. Chem. Chem. Phys..

[36] Nietfeld J.P., Schwiderski R.L., Gonnella T.P., Rasmussen S.C. (2011). Structural effects on the electronic properties of extended fused-ring thieno[3,4-*b*]pyrazine analogues. J. Org. Chem..

[37] Nietfeld J.P., Heth C.L., Rasmussen S.C. (2008). Poly(acenaphtho[1,2-*b*]thieno[3,4-*e*]pyrazine): A new low band gap conjugated polymer. Chem. Commun..

[38] Wen L., Nietfeld J.P., Amb C.M., Rasmussen S.C. (2009). New tunable thieno[3,4-*b*]pyrazine-based materials. Synth. Met..

[39] Karsten B.P., Bijleveld J.C., Viani L., Cornil J., Gierschner J., Janssen R.A.J. (2009). Electronic structure of small band gap oligomers based on cyclopentadithiophenes and acceptor units. J. Mater. Chem..

[40] Becerril H.A., Miyaki N., Tang M.L., Mondal R., Sun Y.-S., Mayer A.C., Parmer J.E., McGehee M.D., Bao Z. (2009). Transistor and solar cell performance of donor-acceptor low bandgap copolymers bearing an acenaphtho[1,2-*b*]thieno[3,4-*e*]pyrazine (ACTP) motif. J. Mater. Chem..

[41] Mondal R., Ko S., Norton J.E., Miyaki N., Becerril H.A., Verploegen E., Toney M.F., Brédas J.-L., McGehee M.D., Bao Z. (2009). Molecular design for improved photovoltaic efficiency: Band gap and absorption coefficient engineering. J. Mater. Chem..

[42] Keshtov M.L., Godovsky D.Y., Khokhlov A.R., Mizobe T., Fujita H., Goto E., Hiyoshi J., Nakamura S., Kawauchi S., Higashihara T. (2015). Synthesis and photovoltaic properties of thieno[3,4-*b*]-pyrazine or dithieno[3′,2′:3,4;2″,3″:5,6]benzo[1,2-*d*]imidazole-containing conjugated Polymers. J. Polym. Sci. A Polym. Chem..

[43] Keshtova M.L., Marochkina D.V., Kochurov V.S., Komarov P.V., Parashchuk D.Y., Trukhanov V.A., Khokhlov A.R. (2014). New narrow-band-gap conjugated copolymers based on benzodithiophene: Synthesis and photovoltaic properties. Polym. Sci. Ser. B.

[44] Pu K., Shuhendler A.J., Jokerst J.V., Mei J., Gambhir S.S., Bao Z., Rao J. (2014). Semiconducting polymer nanoparticles as photoacoustic molecular imaging probes in living mice. Nat. Nanotech..

[45] Petersen M.H., Hagemann O., Nielsen K.T., Jørgensen M., Krebs F.C. (2007). Low band gap poly-thieno-pyrazines for solar cells—Introducing the 11-thia-9,13-diaza-cyclopenta[*b*]triphenylenes. Solar Energy Mater. Sol. Cells.

[46] Mondal R., Miyaki N., Becerril H.A., Norton J.E., Parmer J., Mayer A.C., Tang M.L., Bredas J.-L., McGehee M.D., Bao Z. (2009). Synthesis of acenaphthyl and phenanthrene based fused-aromatic thieno-pyrazine co-polymers for photovoltaic and thin film transistor applications. Chem. Mater..

[47] Mondal R., Ko S., Bao Z. (2010). Fused aromatic thienopyrazines: Structure, properties and function. J. Mater. Chem..

[48] Mondal R., Ko S., Verploegen E., Becerril H.A., Toney M.F., Bao Z. (2011). Side chain engineering of fused aromatic thienopyrazine based low band-gap polymers for enhanced charge carrier mobility. J. Mater. Chem..

[49] Velusamy M., Huang J.-H., Hsu Y.-C., Chou H.-H., Ho K.-C., Wu P.-L., Chang W.-H., Lin J.T., Chu C.-W. (2009). Dibenzo[f,h]thieno[3,4-b] quinoxaline-based small molecules for efficient bulk-hetero-junction solar cells. Org. Lett..

[50] Mak C.S.K., Leung Q.Y., Chan W.K., Djurisic A.B. (2008). Optically tunable intramolecular charge transfer dyes for vacuum deposited bulk heterojunction solar cells. Nanotechnology.

[51] Wang Z., Gao Z., Feng Y., Liu Y., Yang B., Liu D., Lv Y., Lu P., Ma Y. (2013). Highly π-extended polymers based on phenanthro-pyrazine: Synthesis, characterization, theoretical calculation and photovoltaic properties. Polymer.

[52] Nishida J., Murakami S., Tada H., Yamashita Y. (2006). n-Type and ambipolar fet characteristics using pyra-zinophenanthrolines linked with oligothiophenes. Chem. Lett..

[53] Čík G., Krajčovič J., Veis P., Végh D., Šeršen F. (2001). Characterization and properties of the copolymer of dipyrido-[3,2-*a*;2′,3′-*c*]-thien-[3,4-*c*]azine with 3-dodecylthiophene. Synth. Met..

[54] Mondal R., Becerril H.A., Verploegen E., Kim D., Norton J.E., Ko S., Miyaki N., Lee S., Toney M.F., Bredas J.-L. (2010). Thiophene-rich fused-aromatic thienopyrazine acceptor for donor–acceptor low band-gap polymers for OTFT and polymer solar cell applications. J. Mater. Chem..

[55] Schwiderski R.L., Rasmussen S.C. (2013). Synthesis and characterization of thieno[3,4-*b*]pyrazine-based terthienyls: Tunable precursors for low band gap conjugated materials. J. Org. Chem..

[56] Kitamura C., Tanaka S., Yamashita Y. (1996). Design of narrow-bandgap polymers. Syntheses and properties of monomers and polymers containing aromatic-donor and o-quinoid-acceptor units. Chem. Mater..

[57] Wen L., Rasmussen S.C. (2007). Synthesis and structural characterization of 2,5-dihalo-3,4-dinitrothiophenes. J. Chem. Crystallogr..

[58] Sen C.P., Shrestha R.G., Shrestha L.K., Ariga K., Valiyaveettil S. (2015). Low-band-gap BODIPY conjugated copolymers for sensing volatile organic compounds. Chem. Eur. J..

[59] Mikroyannidis J.A., Tsagkournos D.V., Balraju P., Sharma G.D. (2011). Efficient bulk heterojunction solar cells using analternating phenylenevinylene copolymer with dithenyl(thienothiadiazole) segments as donor and PCBM or modified PCBM as acceptor. Sol. Energy Mater. Sol. Cells.

[60] Ogawa K., Stafford J.A., Rothstein S.D., Tallman D.E., Rasmussen S.C. (2005). Nitrogen-functionalized polythiophenes: Potential routes to new low band gap materials. Synth. Met..

[61] Kenning D.D., Mitchell K.A., Calhoun T.R., Funfar M.R., Sattler D.J., Rasmussen S.C. (2002). Thieno-[3,4-*b*]pyrazines: Synthesis, structure, and reactivity. J. Org. Chem..

[62] Rosenblum P. (1968). Solubility in the potassium stannate-potassium hydroxide-water system at 0 to 95.0 °C. Can. J. Chem..

[63] Mo H., Radke K.R., Ogawa K., Heth C.L., Erpelding B.T., Rasmussen S.C. (2010). Solution and solid-state properties of highly fluorescent dithieno[3,2-*b*:2′,3′-*d*]pyrrole-based oligothiophenes. Phys. Chem. Chem. Phys..

[64] Turro N.J. (1991). Modern Molecular Photochemistry.

[65] Birer Ö., Moreschini P., Lehmann K.K., Scoles G. (2007). Electronic spectroscopy of nonalternant hydrocarbons inside helium nanodroplets. J. Phys. Chem. A.

[66] Zhou W.-Q., Peng H.-P., Xu J.-K., Xia H.-Y., Pu S.-Z. (2008). Electrochemical polymerization of phenanthrene in mixed electrolytes of boron trifluoride diethyl etherate and concentrated sulfuric acid. Polym. Int..

[67] Han Z.-B., Cheng X.-N., Chen X.-M. (2005). Effect of the size of aromatic chelate ligands on the frameworks of metal dicarboxylate polymers: From helical chains to 2-D networks. Cryst. Growth Des..

[68] Cardona C.M., Li W., Kaifer A.E., David Stockdale D., Bazan G.C. (2011). Electrochemical considerations for determining absolute frontier orbital energy levels of conjugated polymers for solar cell applications. Adv. Mater..

[69] Kissinger P.T., Preddy C.R., Shoup R.E., Heineman W.R., Kissinger P.T., Heineman W.R. (1996). Fundametal concepts of analytical electrochemistry. Laboratory Techniques in Electroanalytical Chemistry.

[70] Waltman R.J., Diaz A.F., Bargon J. (1984). Electroactive properties of polyaromatic molecules. J. Electrochem. Soc..

[71] Waltman R.J., Bargon J. (1986). Electrically conducting polymers: A review of the electropolymerization reaction, of the effects of chemical structure on polymer film properties, and of applications towards technology. Can. J. Chem..

[72] Gurunathan K., Murugan A.V., Marimuthu R., Mulik U.P., Amalnerkar D.P. (1999). Electrochemically synthesised conducting polymeric materials for applications towards technology in electronics, optoelectronics and energy storage devices. Mater. Chem. Phys..

[73] Larson R.C., Iwamoto R.T., Adams R.N. (1961). Reference electrodes for voltammetry in acetonitrile. Anal. Chim. Acta.

[74] Turro N.J. (1991). Modern Molecular Photochemistry.

