# Supplementary material for: Synthesis, Characterization, and Electropolymerization of Extended Fused-Ring Thieno[3,4-b]pyrazine-Based Terthienyls
Source: Materials (Basel). 2016 May 26;9(6):404. doi: 10.3390/ma9060404 (PMC5456834; doi:10.3390/ma9060404)
Supplement: Supplementary file 1 [file materials-09-00404-s001.pdf]

Supplementary Materials: Synthesis, Characterization, and Electropolymerization of Extended Fused-Ring Thieno[3,4-*b*]pyrazine-Based Terthienyls

Kristine L. Konkol, Ryan L. Schwiderski and Seth C. Rasmussen

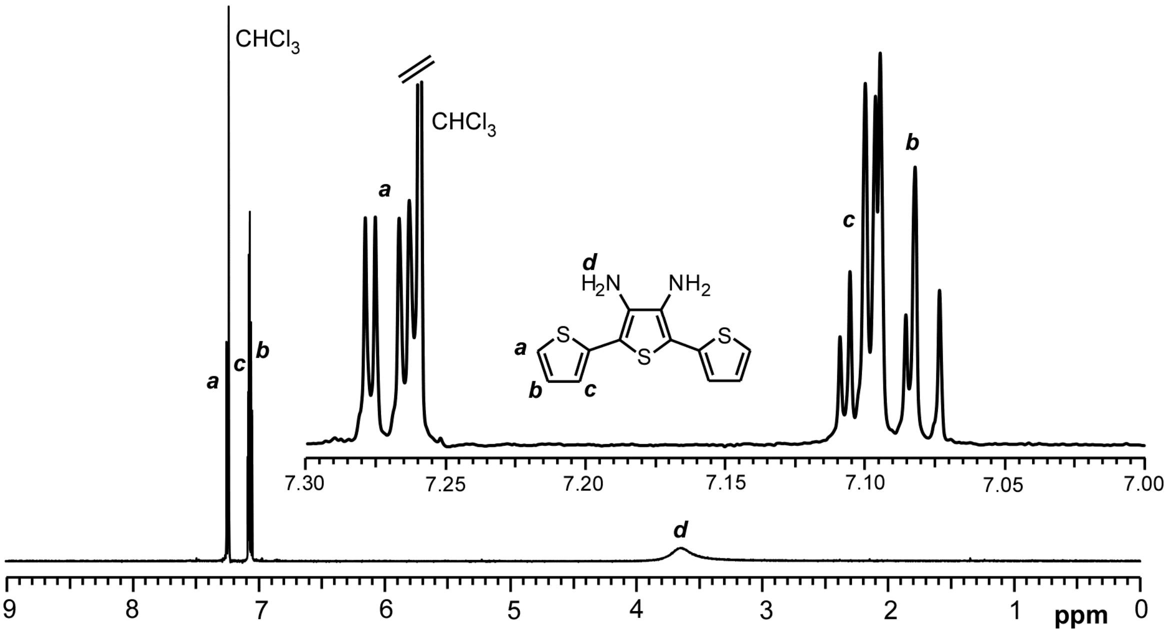

Figure S1. ^{1}H NMR Spectrum of Compound **10**.

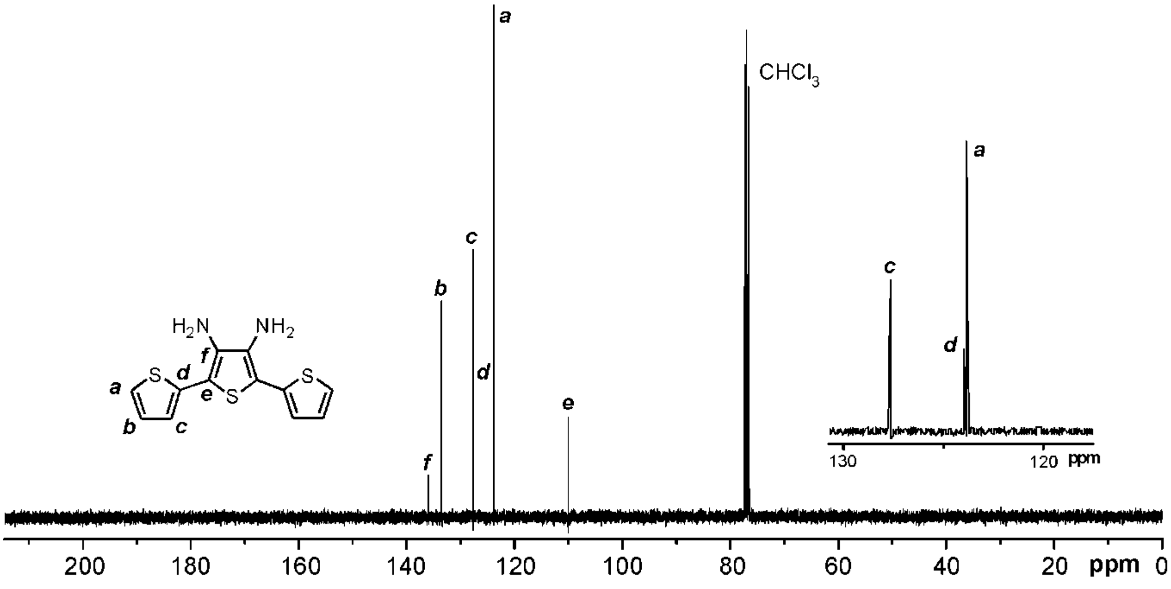

Figure S2. ^{13}C NMR Spectrum of Compound **10**.

Modeling of 2nd order coupling effects in Compound 10. To verify the assignment of peaks near 7.1 ppm in the ^{1}H NMR spectrum of compound **10** as two strongly coupled doublet-of-doublets, the experimentally determined coupling constants and chemical shifts were used to simulate the NMR spectrum. This was accomplished using a freely available NMR simulator [1] which could accurately model the 2nd order effects inherent from the close spacing of the coupled multiplets [2]. The values used for the modeling are as follows:

Spin system: ABC

delta 1 (ppm): 7.1018

delta 2 (ppm): 7.0848 J1-2 (Hz): 3.65

delta 3 (ppm): 7.2714 J1-3 (Hz): 1.44 J2-3 (Hz): 4.84

From: 7.0 to: 7.3 ppm

Field strength: 400 MHz

Line width: 0.5 Hz

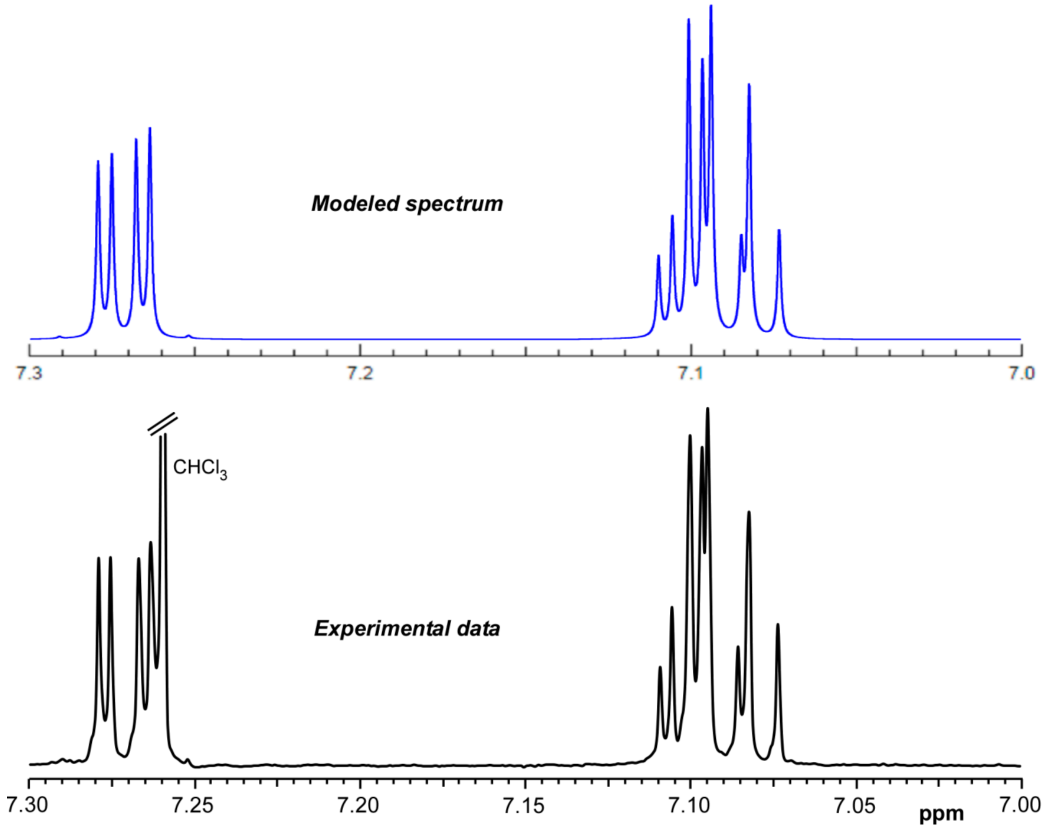

Figure S3. Comparison of results from modeling of 2nd order coupling effects in the ^{1}H NMR spectrum of **10**.

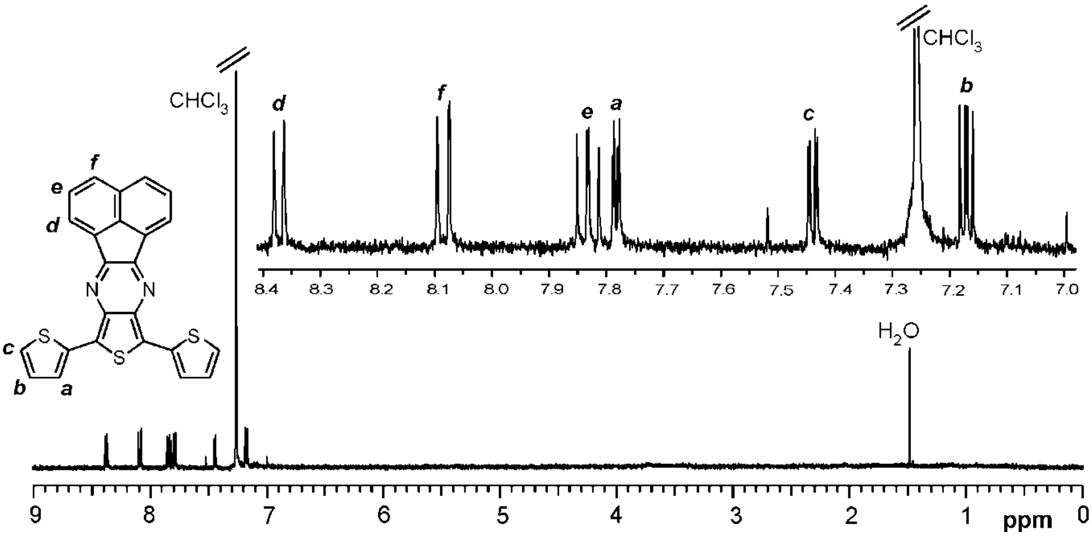

Figure S4. ^{1}H NMR Spectrum of Compound **T3**.

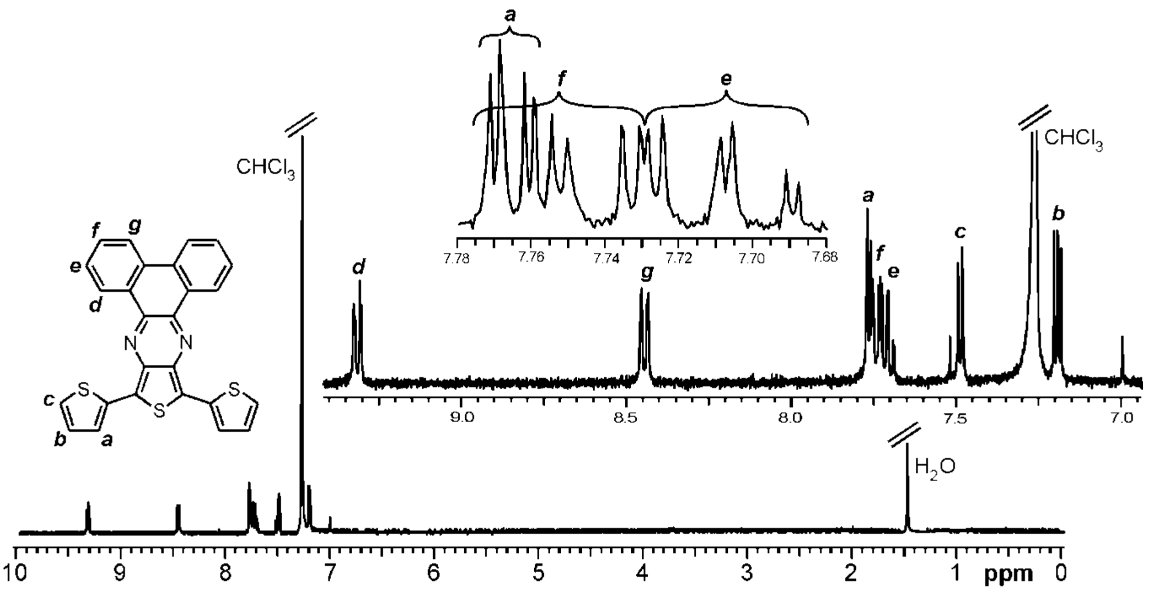

Figure S5. ^{1}H NMR Spectrum of Compound **T4**.

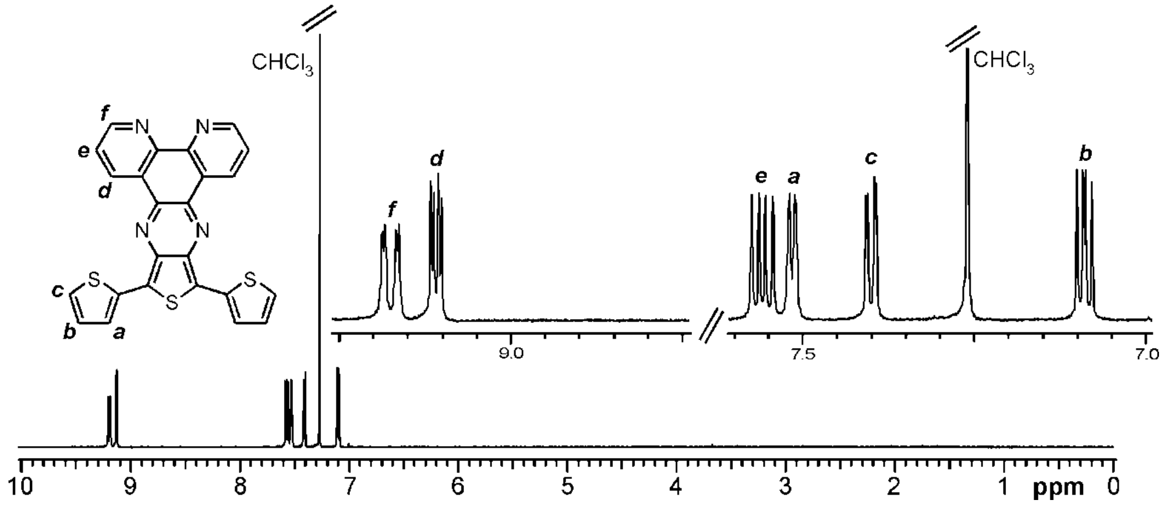

Figure S6. ^{1}H NMR Spectrum of Compound **T5**.

References

1. Institute of Chemical Sciences and Engineering. Tools for NMR Spectroscopists. Available online: http://www.nmrdb.org/simulator/index.shtml?v=v2.34.1 (accessed on 22 April 2016).
2. Castillo, A.M.; Patiny, L.; Wist, J. Fast and accurate algorithm for the simulation of NMR for large spin systems. *J. Mag. Res.* **2011**, *209*, 123–130.
